# Positive selection on two mitochondrial coding genes and adaptation signals in hares (genus *Lepus*) from China

**DOI:** 10.1186/s12862-021-01832-7

**Published:** 2021-05-26

**Authors:** Asma Awadi, Hichem Ben Slimen, Helmut Schaschl, Felix Knauer, Franz Suchentrunk

**Affiliations:** 1 Laboratory of Functional Physiology and Valorization of Bioresources, Higher Institute of Biotechnology of Beja, University of Jendouba, Jendouba, Tunisia; 2 Department of Evolutionary Anthropology, University of Vienna, Althanstrasse 14, 1090 Vienna, Austria; 3 Research Institute of Wildlife Ecology, University of Veterinary Medicine Vienna, Savoyenstrasse 1, 1160 Vienna, Austria

**Keywords:** Mitochondrial DNA, Positive selection, Purifying selection, Environmental variation, Protein modelling, Hares, China

## Abstract

**Background:**

Animal mitochondria play a central role in energy production in the cells through the oxidative phosphorylation (OXPHOS) pathway. Recent studies of selection on different mitochondrial OXPHOS genes have revealed the adaptive implications of amino acid changes in these subunits. In hares, climatic variation and/or introgression were suggested to be at the origin of such adaptation. Here we looked for evidence of positive selection in three mitochondrial OXPHOS genes, using tests of selection, protein structure modelling and effects of amino acid substitutions on the protein function and stability. We also used statistical models to test for climate and introgression effects on sites under positive selection.

**Results:**

Our results revealed seven sites under positive selection in *ND4* and three sites in *Cytb*. However, no sites under positive selection were observed in the *COX1* gene. All three subunits presented a high number of codons under negative selection. Sites under positive selection were mapped on the tridimensional structure of the predicted models for the respective mitochondrial subunit. Of the ten amino acid replacements inferred to have evolved under positive selection for both subunits, six were located in the transmembrane domain. On the other hand, three codons were identified as sites lining proton translocation channels. Furthermore, four codons were identified as destabilizing with a significant variation of Δ vibrational entropy energy between wild and mutant type. Moreover, our PROVEAN analysis suggested that among all positively selected sites two fixed amino acid replacements altered the protein functioning. Our statistical models indicated significant effects of climate on the presence of ND4 and Cytb protein variants, but no effect by trans-specific mitochondrial DNA introgression, which is not uncommon in a number of hare species.

**Conclusions:**

Positive selection was observed in several codons in two OXPHOS genes. We found that substitutions in the positively selected codons have structural and functional impacts on the encoded proteins. Our results are concordantly suggesting that adaptations have strongly affected the evolution of mtDNA of hare species with potential effects on the protein function. Environmental/climatic changes appear to be a major trigger of this adaptation, whereas trans-specific introgressive hybridization seems to play no major role for the occurrence of protein variants.

## Background

Animal mitochondria are cell organelles which play a central role in ATP production in the cells through oxidative phosphorylation (OXPHOS) [1, 2]. Recent publications revealed additional functions of mitochondria such as regulation of apoptosis, intracellular macromolecule assembly and immunological cross-talk [3]. In most animals the mtDNA is a short, circular molecule that contains about 13 protein-coding genes involved in the OXPHOS process [1]. Some of the latter genes have been widely used in population genetic studies, phylogeographical and phylogenetic reconstructions and were for a long period considered evolving as a strictly neutral genetic marker. However, the assumption of non-neutral evolution of mtDNA can interfere with historical inferences in population and evolutionary biology [4]. Indeed, mitochondrial genes encoding for OXPHOS subunits are expected to be under selection, because variations in these genes may affect the organism fitness by directly influencing the metabolic performance with potential effects on the immunity [5, 6]. Therefore, mtDNA-encoded OXPHOS genes present very good study systems for understanding the evolution of adaptive traits in species. Over the past two decades, several tests of selection at the molecular level have been successfully used to detect signatures of adaptive evolution in mtDNA genes [7–12]. Evidence of adaptive selection was also obtained from experimental studies that demonstrated that the intra-specific genetic variation that exists within the mitochondrial genome commonly affects the expression of phenotypic traits of morphology, metabolism, and life history [13–17]. The “mitochondrial climatic adaptation hypothesis” was often used to explain selection on mtDNA. This hypothesis suggests that functional variation between mtDNA haplotypes plays an important role in shaping the genetic adaptation of populations to the temperatures of their environments [18]. The basis of this hypothesis is that mitochondrial genes encode multiple subunits in different enzyme complexes responsible for mitochondrial respiration, and these enzymatic processes are highly temperature sensitive [17].

In China, hares (*Lepus*) are distributed from the Qinghai-Tibetan Plateau to near sea level, and from the cold north of the interior mainland to the tropic island of Hainan [19]. Their evolutionary history is not fully understood, partly due to uncertain taxonomic classification, partly due to a lack of comprehensive data on molecular variability of taxa [19]. The taxonomic uncertainty is due to high phenotypic variation in contrasting environments and to frequent introgressive hybridization between the different Chinese hare species [19], and also because of missing molecular phylogenetic and population genetic studies including Chinese taxa and important forms from outside of China. In their phylogenetic study of 116 specimens from China assigned to eight species, Liu et al. [19] revealed eight lineages grouped into five major clades for mitochondrial genes, with uncorrected pairwise p-distances ranging from 2.2–8.5% for *cytb* sequences to 5.9–15.3% for control region sequences, respectively. The phylogenetic model of a nuclear gene fragment (MGF – stem cell factor) of a slightly bigger sample of Chinese specimens developed by the same authors [19], however, yielded 72 haplotypes arranged in only six lineages partitioned into two major phylogenetic complexes (a “*L.* *capensis*” and a “*L.* *sinensis* group”). For one species (*L.* *mandshuricus*), no species-specific mitochondrial lineage was revealed, seemingly representing a case of “mitochondrial capture”. Moreover, whereas mitochondrial sequences indicated likely only unidirectional introgression among species, the nuclear sequences indicated bidirectional introgression among *L.* *yarkandensis* and *L.* *capensis*.

Indeed, hares and jackrabbits (genus *Lepus*) form a highly polymorphic group of closely related species displaying a young radiation history with interspecific mtDNA introgression described both in current hybrid zones [e.g., 20, 21] and in areas of ancient contact between species [21, 22]. They are widely distributed in many forms across large parts of the world and numerous contrasting environments [e.g., 23]. Intraspecific phenotypic variation was observed even within relatively small ranges [e.g., 9, 24, 25]. Such characteristics make them an ideal “natural experiment” to study genetic adaptation to different climatic/environmental conditions. Correspondingly, several studies have detected positive selection in mtDNA-encoded OXPHOS genes of the genus *Lepus* in the context of environmental and climate conditions [9, 10, 26, 27]. Moreover, Melo-Ferreira et al. [26] suggested that adaptation may have influenced the occurrence and consequences of the many reported cases of massive mtDNA introgression. Indeed, Canestrelli et al. [28] suggested that hybridization between individuals from different locally-adapted populations, when they come into secondary contact, enables selection of novel mito-nuclear genotypes that might be better suited to a new or changing environment.

In this study, we tested for signatures of natural selection on three complete mtOXPHOS genes (cytochrome oxidase 1 (*COX1*), cytochrome B (*Cytb*), and *NADH* *dehydrogenase* *4* *(ND4)*) retrieved from GenBank [19], from eight Chinese hare species occupying different habitats. These genes are likely to be a target for selection due to their essential function in energy production. Indeed, earlier studies on these subunits have shown that they are under different selective pressures. In fact, *ND4* showed the highest number of positively selected sites contrary to *COX1* that exhibited the lowest number among all mtOXPHOS genes tested in hares by Melo-Ferreira et al. [26]. On the other hand, positively selected sites of *Cytb* were correlated with temperature adaptation on the marine mammal killer whale and in the European anchovy [29]. Our specific aims were (i) to test for positive selection on protein variants of the studied genes, (ii) to test whether positive selection was associated with climate variation and/or introgressed lineages, and might thus reflect adaptation, and (iii) to test whether amino-acid changes had an impact on the biological function of the encoded protein variants.

## Results

### Evidence of natural selection

We used different methods to assess positive and negative selection affecting specific codons in the mtOXPHOS genes. All sites under positive selection as suggested by the diverse methods implemented are summarized in Table 1; however, only sites with more than one method suggesting positive selection are shown (as recommended by [26]).

**Table 1:** Results of selection tests according to six different methods (only sites with suggestion of selection by two or more methods are shown); x—positive selection as indicated by a significant selection test signal

	Site	Ancestral	Aminoacid change	PAML	DATAMONKEY				TreeSAAP (Proprety)	Provean	Domain	DYNAMUT	
					SLAC	FEL	MEME	FUBAR				ΔΔG (kcal/mol)	action
ND4	**29**	**V**	**A**	**x**	**–**	**–**	**–**	**–**	**x (*pK’*)**	**− 0.001**	**Trans**	**− 0.127**	**Destabilizing**
	101	S	A	**–**	**–**		x	**–**	x (*Pα*)	**− **0.547	Trans	1.098	Stabilizing
	185	H	S	**–**	x	x	x	x	**–**	0.498	Inter	0.461	Stabilizing
	**187**	**L**	**P**	**–**	**x**	**x**			**x (*Pα*)**	**− 0.085**	**Inter**	**− 0.076**	**Destabilizing**
	**246**	**I**	**V**	**–**	**–**	**x**	**–**	**–**	**x (*pK’*)**	**− 0.627**	**Trans**	**− 0.643**	**Destabilizing**
	**305**	**T**	**S**	**–**		**x**	**x**	**x**	**–**	**− 3.444**	**Trans**	**0.052**	**Stabilizing**
	**425**	**N**	**V**	**–**		**x**	**x**	**–**	**–**	**− 3.685**	**Matrix**	**0.01**	**Stabilizing**
CytB	23	T	A	**–**	x	x	**–**	**–**	x (*Pα*)	**− **1.406	Matrix	0.518	Stabilizing
	194	M	L	**–**	**–**	**–**	x	**–**	x (*pK’*)	**− **0.045	Trans	0.274	Stabilizing
	**356**	**I**	**V**	**–**	**–**	**x**	**–**	**–**	**x (*pK’*)**	**0.002**	**Trans**	**− 0.083**	**Destabilizing**

Replacements inferred to be function-altering by PROVEAN and DYNAMUT are indicated in bold

Equil. Const. – ioniza., COOH (*pK*’); α − helical tendencies (Pα)

In the site model analyses from CODEML (Table 2) the null model was rejected in all pairwise comparison for the *ND4* gene variants indicating that neutrality can be rejected and confirmed the existence of variable ω values across sites. These results suggested also that the *ND4* gene was globally evolving under negative constraints, with a few percent of codons evolving under neutrality or positive selection. Indeed, the codon-based test implemented in PAML revealed a unique codon under positive selection in *ND4*. The Bayes Empirical Bayes (BEB) procedure, for both model M2a and M8, identified codon 29 in *ND4* under positive selection. The CODEML analyses for *Cytb* showed that the alternative models with two classes of sites, ω = 1 and ω < 1 (model M1a) or three fixed classes of ω (ω = 1, ω < 1 and ω > 1) fitted better the data than model M0. However, no sites under positive selection were detected. In contrast, the CODEML analyses for *COX1* showed that null models fitted better the data than models with positive selection indicating that this gene was evolving under neutrality.

**Table 2:** Results of PAML analyses testing for selection on the three mitochondrial subunits

Gene	Model	*p*	Parameter estimates	Log likelihood	*Sites*	Model comparison	*p* *(ΔLRT)*
*ND4*	M0 (one-ratio)	1	ω0 = 0.0372	− 7708.624	–	M0 vs M1a M0 vs M3 M1a vs M2a M7 vs M8	< 0.001 < 0.001 < 0.001 < 0.001
	M1 (neutral)	2	ω0 = 0.0074 p0 = 0.9469 ω1 = 1.000 p1 = 0.0531	− 7645.299	–		
	M2 (selection)	4	ω0 = 0.0072 p0 = 0.947 ω1 = 1.0000 p1 = 0.0523 ω2 = 2.1673 p2 = 0.0002	− 7504.371	29		
	M3 (discrete)	5	ω0 = 0.0063 p0 = 0.9425 ω1 = 0.6554 p1 = 0.0575 ω2 = 217.8820 p2 = 0.0000	− 7497.046	–		
	M7 (beta)	2	p = 0.0062 q = 0.005	− 8315.174	–		
	M8 (beta&ω)	4	p0 = 0.9658 p = 0.0631 q = 2.7963 (p1 = 0.0342) ω = 1.0000	− 7499.443	29		
*CytB*	M0 (one-ratio)	1	ω0 = 0.0320	− 5881.359	–	M0 vs M1a M0 vs M3 M1a vs M2a M7 vs M8	< 0.001 < 0.001 > 0.05 > 0.05
	M1 (neutral)	2	ω0 = 0.0115 p0 = 0.9660 ω1 = 1.000 p1 = 0.0340	− 5774.833	–		
	M2 (selection)	4	ω0 = 0.0115 p0 = 0.9660 ω1 = 1.000 p1 = 0.0340 ω2 = 14.7728 p2 = 0.0000	− 5785.211	–		
	M3 (discrete)	5	ω0 = 0.0072 p0 = 0.9469 ω1 = 0.4605 p1 = 0.0531 ω2 = 14.1110 p2 = 0.0000	− 5785.565	–		
	M7 (beta)	2	p = 0.0165 q = 0.0239	− 5908.619	–		
	M8 (beta&ω)	4	p0 = 0.9999 p = 0.0590 q = 1.0510 (p1 = 0.00001) ω = 6.0347	− 5911.451	–		
*cox1*	M0 (one-ratio)	1	ω0 = 0.0047	− 5912.722	–	M0 vs M1a M0 vs M3 M1a vs M2a M7 vs M8	> 0.05 > 0.05 > 0.05 < 0.001
	M1 (neutral)	2	ω0 = 0.0039 p0 = 0.9974 ω1 = 1.000 p1 = 0.0027	− 5910.938	–		
	M2 (selection)	4	ω0 = 0.0039 p0 = 0.9974 ω1 = 1.000 p1 = 0.0026 ω2 = 34.4363 p2 = 0.0000	− 5911.120	–		
	M3 (discrete)	5	ω0 = 0.0038 p0 = 0.9966 ω1 = 0.5872 p1 = 0.0034 ω2 = 6.8990 p2 = 0.0000	− 5911.043	–		
	M7 (beta)	2	p = 0.0101 q = 0.0086	− 6208.708	–		
	M8 (beta&ω)	4	p0 = 0.9999 p = 0.0564 q = 6.7184 (p1 = 0.00001) ω = 6.2162	− 5911.451	–		

The CODEML results were complemented by those of DATAMONKEY, which allowed identifying codons under negative selection in addition to those under positive selection. For *ND4*, six codon positions (101, 185, 187, 246, 305 and 425) were suggested to be under positive selection by our DATAMONKEY tests. Only codon position 185 was confirmed by all four applied datamonkey tests, while codon position 305 was confirmed by three tests. For *Cytb*, positive selection was observed at codon position 23, 194, and 306. No evidence of positive selection was observed in the *COX1* gene as suggested by the different DATAMONKEY tests.

Furthermore, according to the SLAC and FEL analyses (on the DATAMONKEY web server), all three mitochondrial subunits (*COX1*, *Cytb,* *ND4*) presented a high percentage of codons under negative selection with the *ND4* subunit from complex I showing the highest number of codons under negative selection (169 and 294 sites for SLAC and FEL, respectively). *Cytb* from complex III (122 and 232 sites for SLAC and FEL, respectively) and the *COX1* subunits from complex IV (142 and 207 sites for SLAC and FEL, respectively) also revealed a high number of codons under purifying selection.

Finally, several codons (26 and 28 for *ND4* and *Cytb*, respectively) were identified as possibly under positive selection when using TreeSAAP. For ND4 protein variants, three radical physicochemical property changes were suggested by TreeSAAP: propriety Equilibrium constant (ionization of COOH), Alpha-helical tendencies and Isoelectric point. Only the first two were altered in *Cytb*.

### Homology modeling and mutation effects

In order to understand how the sites under positive selection influence the protein structure and/or function of ND4 and Cytb, protein models for both mitochondrial subunits were generated using the ovine (5LNK.1.K) and the bovine (6HAW.1.C) structure as a template. The sequence identity was 81.26% and the GMQE (Global Model Quality Estimate) was 0.97 for *ND4*, and 85.98% and 0.98 for *Cytb*, respectively.

**Fig. 1:**
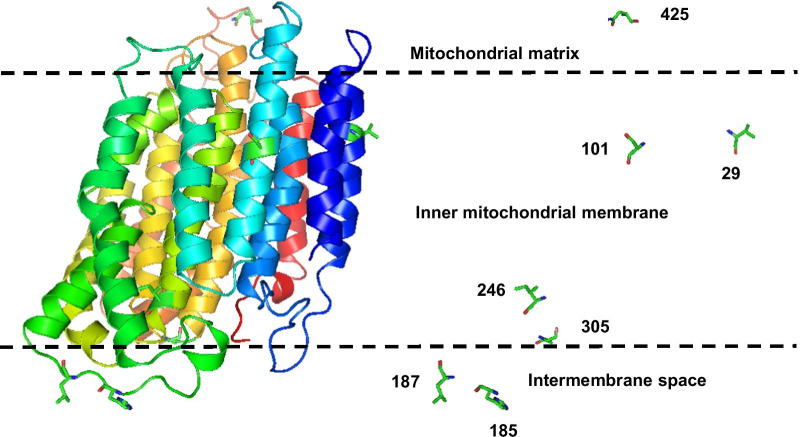
Mapping of the positively selected sites on the 3D structure of the obtained model for ND4. On the right hand side, positively selected sites are represented as sticks. The mitochondrial inner membrane (MIM), as suggested by MEMEMBED [42] is roughly delineated with the two parallel dashed lines

**Fig. 2:**
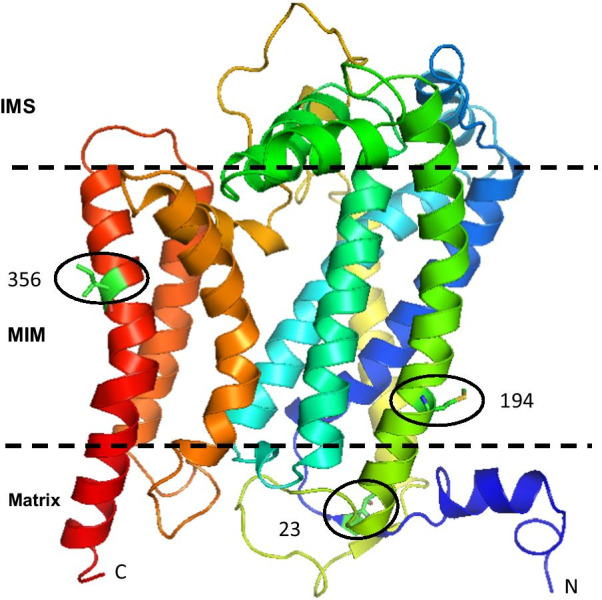
Mapping of the positively selected sites on the 3D structure of the obtained model for Cytb. The subunit is oriented with the matrix side at the bottom of the page and the intermembrane space (IMS) on the top. The mitochondrial inner membrane (MIM), as suggested by MEMEMBED [42] is roughly delineated with the two parallel dashed lines

Structurally, ND4 and Cytb displayed a conserved secondary arrangement when compared to their ovine and bovine counterparts, respectively. Indeed, the *Lepus* ND4 protein model displayed a secondary structure that consisted of 65.4% alpha helices, 2% 3_10_ helices, 1.1% pi helices and 8.7% turns (Fig. 1). These domains were arranged in 12 transmembrane, 7 cytoplasmic and 6 extracellular regions. The *Cytb* model was composed of 62.7% alpha helices, 6.6% 3_10_ helices, 2.6% pi helices, 1.6% beta strands and 9.3% turns (Fig. 2). These domains were arranged in 8 transmembrane, 5 cytoplamsic and 4 extracellular regions. N- and C-terminal regions of both subunits did represent a cytoplasmic domain location. Domains of *ND4* and *Cytb* subunits were arranged in 14 and 8 TM helices, respectively, as indicated by the superimposition with the 3D structure of the ovine 5LNK.1.K (a counterpart of ND4) and the bovine 6HAW.1.C (a counterpart of Cytb).

The quality of the 3D models was evaluated via the Ramachandran plot using the PROCHECK software and the ERRAT server. For *ND4*, the Ramachandran plot for the predicted model revealed that 90.5% of residues were in the most favorable region, while 9.0% were in the allowed region. The overall quality factor predicted by the ERRAT server was 90.687. For *Cytb*, the Ramachandran plot for the predicted model revealed that 93.3% of residues were in the most favorable region, while 6.4% were in the allowed region. The overall quality factor predicted by the ERRAT server was 96.216.

The positively selected sites of both genes were mapped onto the predicted *Lepus* 3D structure allowing to localize them within one of three mitochondrial domains, namely the matrix, or the inner membrane (i.e. transmembrane), or between inner and outer membrane (i.e. intermembrane). Of the ten amino acid replacements inferred to have evolved under positive selection for both subunits, six (sites 29, 101, 246 and 305 for *ND4*; sites 194 and 356 for *Cytb*) were located in the transmembrane domain, two (sites 185 and 187 of *ND4*) in the intermembrane space and two (sites ND4-425 and Cytb-23) in the mitochondrial matrix (Table 1, Figs. 1 and 2). On the other hand, positions 101 and 305 of *ND4* and 194 of *Cytb* were identified by MEMSAT-SVM as sites lining proton translocation channels. Moreover, site 23 of the *Cytb* subunit was the only positively selected site involved in interactions between subunits, namely between *Cytb* and subunit 7 of the Cytochrome bc1 complex encoded by the nuclear UQCRB gene.

**Fig. 3:**
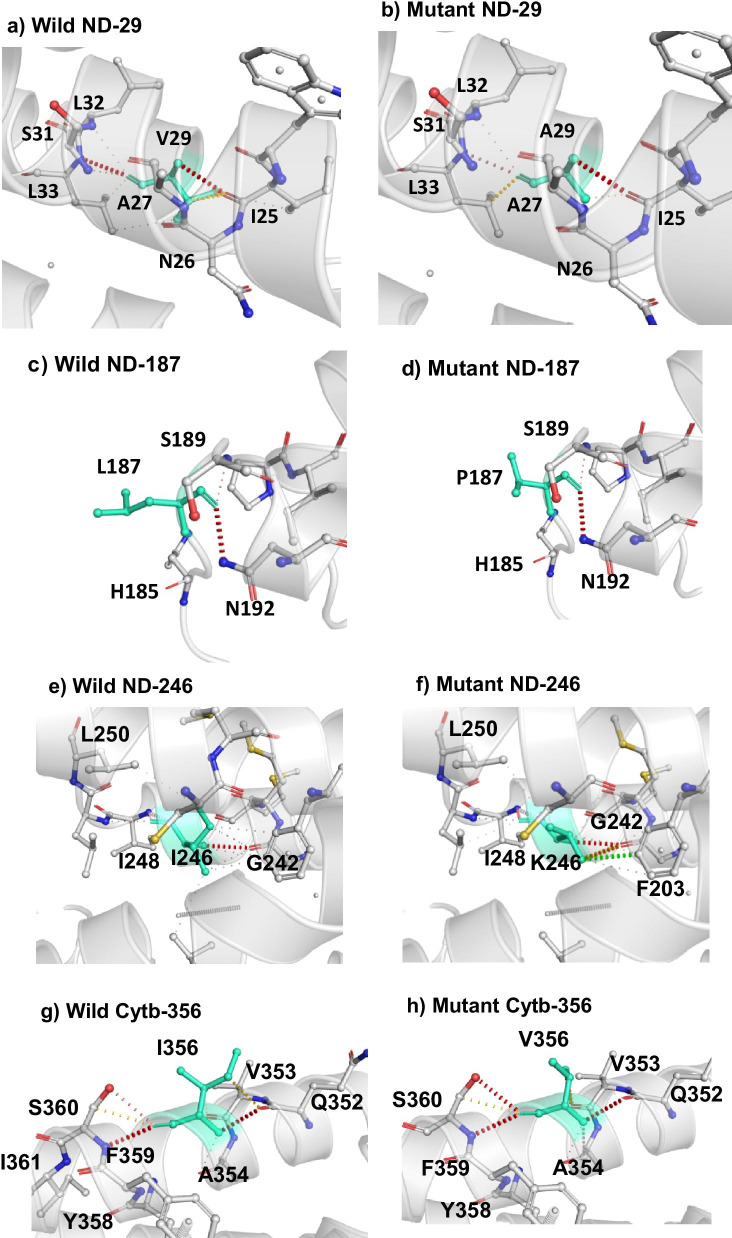
Interatomic Interactions predictions of wild-type and mutant residues as obtained from DynaMut. Wild-type and mutant residues are coloured in light-green and are also represented as sticks. Dashed lines indicated different types of interactions, red: Hydrogen bonds, green: Hydrophobic contacts, yellow: Ionic interactions

Among the ten candidate sites for positive selection, four (ND4-29, ND4-187, ND4-246, Cytb-356) were identified as destabilizing with Δ vibrational entropy energy between wild and mutant type indicating an increase of molecule flexibility for ND4-187 while the three other sites showed a decrease of molecule flexibility (Table 1, Fig. 3). Finally, the PROVEAN analysis suggested that among all positively selected sites two fixed amino acid replacements altered the protein functioning (Table 1). These included the replacements T305S and N425V of ND4. Both amino acid substitutions were observed only in *L.* *capensis*. Notably, among all deleterious mutations detected by PROVEAN, five and six were concordantly destabilizing for Cytb and ND4 proteins, respectively, as revealed by DYNAMUT.

Among 54 non synonymous mutations of the *ND4* gene, 21 were destabilizing with Δ vibrational entropy energy between wild and mutant type indicating an increase of molecule flexibility for 15 mutations and a decrease of molecule flexibility in six cases. For *Cytb*, among the 42 non synonymous mutations, 22 were destabilizing with Δ vibrational entropy energy between wild and mutant type indicating an increase of molecule flexibility for 14 mutations and decrease of molecule flexibility in 8 cases. Only sites under positive selection in both genes are shown in Figs. 1 and 2 and their predicted actions are reported in Table 1 and Fig. 3.

### PCA of climate data and statistical models of occurrence of ND4 and Cytb protein variants

The PCA resulted in four principal components (climate factors) that reflected altogether 94.7% of the original climate variables. Climate factor 1 represented 44.7% of the variable variance. It could be interpreted as reflecting annual temperature and particularly also during the coldest and driest period of the year, as well as precipitation during that period. Climate factor 2 summarized 32.7% of the initial data variability and was interpreted as a sole precipitation factor, specifically for the wettest and warmest period of the year. Climate factor 3 (reflecting 12.4% of initial variability) was somewhat difficult to interpret, due to its relatively low variable loadings (all < 0.6); however, the highest variable loadings suggested at least to some extent an interpretation as a factor of temperature seasonality, specifically also of high ambient temperature during the warmest and wettest period of the year. Finally, climate factor 4, summarizing 5.9% of the data variability, could be interpreted as reflecting mostly precipitation seasonality.

The multinomial model runs indicated statistically meaningful effects of the climate factor 1, climate factor 2 and climate factor 3 on the presence of ND4 protein variants. For Cytb protein variants, the logistic glm runs indicated statistically meaningful effects of climate fac.1 and climate fac.3. The relative variable importance values (RVI) of the explanatory variables and factors of the models of the occurrence of ND4 and the Cytb protein variants, as obtained from model averaging, are given in Table 3. Notably, for both genes no introgression effect on the occurrence of the tested protein variants was revealed by our statistical models.

**Table 3:** Variable importance values for the factors considered in the models of amino acid substitutions (values above 0.7—0.8 indicate significant factor effects)

	Climate factor 1	Climate factor 2	Climate factor 3	Climate factor 4	introgression	co-occurring protein variant^a^
*MT-ND4* models	1	1	1	0.1	0.3	< 0.01
*MT-Cytb* models	0.98	0.32	0.93	0.28	0.26	–

^a^ i.e., for the Cytb loucs; for the *MT-Cytb* models there was no chance to account for the co-occurring protein variants at the *MT-ND4* locus, because of too many variants for that latter locus

## Discussion

In our current study, we described and provided evidence of positive selection acting on the two mitochondrial coding genes *ND4* and *Cytb* in eight hare species (genus *Lepus*) from China using a wide range of selection tests, protein structure modeling and analysis of mutation effects. Positive selection was earlier recorded on different mitochondrial OXPHOS genes in a wide range of animal species (see [30] for an overview). Particularly, several studies have focused on mtDNA evolution driven by natural selection in hares and jackrabbits (genus *Lepus*) during the last years. The analyses of eleven mitogenomes of different hare species of temperate and arctic origins by Melo-Ferreira et al. [26] have suggested positive selection in several codons of genes of the mtOXPHOS complexes. However, the structure and the physicochemical properties of the encoded proteins seemed to be not affected by these amino-acids substitutions. The second evidence of occurrence of positive selection on mtOXPHOS genes was observed in *ATP6* and *ND2* sequences of hares (*Lepus* *capensis*) from Tunisia where they were continuously distributed across a steep ecological gradient and exhibited significantly varying ATP6 and ND2 protein frequencies despite high gene flow in putatively neutrally evolving markers [9]. The positive selection signals were interpreted as reflecting adaptation of those hares to the different environmental conditions along the ecological cline in Tunisia [9]. The same two genes studied in 22 hare species distributed across the whole world [10] showed also positive selection with a significant climate effect for ND2 protein variants. Recently, Stefanović et al. [27] demonstrated that only one codon position has evolved under positive selection in the NADH dehydrogenase subunit 6 (*mtND6*) gene in brown hares (*Lepus* *europaeus*) from Europe and the Middle East. The authors suggested that two (D and F) among all observed protein variants were significantly favored under certain precipitation conditions, as proved by statistical models.

Given that the oxidative phosphorylation process produces 95% of cellular energy, it is not surprising that genes encoding for OXPHOS subunits are under adaptive selection. Giannoulis et al. [5] suggested that variations in these genes would directly influence the metabolic performance which may in turn affect the fitness of an organism. Generally, variation in mitochondrial OXPHOS genes may convey a signal of adaptation to environmental, particularly climatic, conditions [e.g., 6, 8–10, 26, 31, 32]. We have used several molecular-statistical approaches to assess the importance of natural selection in the evolution of the studied three mtDNA subunits in hares from China and to disentangle potential effects of climate conditions and evolutionary history of the species on selection acting on those genes. Our site and codon model results (Table 2) indicates that the *ND4* and *Cytb* genes in the studied hares are broadly evolving under negative constraints, i.e., purifying selection, with a small percentage of codons evolving under neutrality or positive selection, reinforcing their crucial and conserved role for the body energy production in mammals. This is also in agreement with the general tendency of the mitochondrial genome evolution in vertebrates (see e.g., [26]) where several studies identified purifying selection as the predominant force shaping the evolution of mtDNA with only few sites and loci under positive selection [26, 33–35]. Indeed, Tomasco and Lessa [36] suggested that, due to the functional importance of mitochondrial genes, purifying selection would be the dominant force in their evolution, preventing fixation of detrimental mutations.

Currently, evidence of positive selection was detected only for the *ND4* and *Cytb* genes, but not for the *COX1* gene. Evidence of positive selection was found by CODEML, the site specific tests implemented in Datamonkey (FEL, SLAC, MEME and FUBAR), and by the Tree-SAAP analyses. Overall, ten codons were inferred to be under positive selection for both genes as suggested by more than one of the tests used in this study. Such positively selected site variation can be striking in both subunits as such amino acid changes might be critical for the optimal molecular function of *ND4* as electron transporter and as regards its structural role between the membrane-embedded and peripheral arms of the complex I [11]. The observed amino acid changes may also be important to the catalytic activity (cytochrome c reduction) of the *Cytb*, which is the only mtDNA-derived subunit of Complex III. Notably, among the currently identified positions under positive selection, positions ND4-29, ND4-187 and ND4-246 were also suggested to be under positive selection in the (much smaller) sample of hare sequences studied by Melo-Ferreira et al. [26].

We have placed special attention to the candidate sites for positive selection in order to assess the potential impact of the observed amino acid substitutions considering their location relative to known functional domains of the proteins and the physicochemical properties of the amino acid, such as size and charge. Sixty percent of the sites under positive selection were located in the transmembrane regions. Moreover, among the positively selected sites, we observed distinct amino acid substitutions at the sites ND4-101, ND4-305, and Cytb-194, which are suggested to be lined up along the proton translocation channel. Indeed, the amino acids located in the transmembrane domain of all OXPHOS complexes were suggested to play essential structural and functional roles related to the proton transport across the membrane [37–40]. Subunits *ND2*, *ND4*, and *ND5* of the mammalian OXPHOS complex I were suggested to be proton-pumping devices which are related to Na+/H+ antiporters of the Mrp family [11]. In cytochrome b (Complex III), the transmembrane domain is often functionally conserved, being involved in the creation of the proton gradient and the transfer of electrons to Complex IV [41]. Consequently, for mammals and other vertebrates, mutations in the mtDNA subunits may interfere with the efficiency of the proton-pumping process and could hinder or improve the proton translocation [11, 41]. These domains are less variable and likely constrained by stronger purifying selection, so amino acid replacements in these domains may suggest a change in OXPHOS protein function that could be subject to positive selection [11, 41–43].

The analyses of the vibrational entropy change upon mutation by the DYNAMUT software identified three amino acid replacements in ND4 and one replacement in Cytb that affect protein stability. On the other hand, among all positively selected sites, two in ND4 were suggested to be deleterious as indicated by the PROVEAN software. Notably, for the sites *ND4*-425 and *Cytb*-356 disease related mutations (LHON disease) were described in humans [44, 45]. Azevedo et al. [46] suggested that the deleterious effect of a mutation can be compensated by a second-site interacting residue which explains why mutations that are deleterious in some species are tolerated in phylogenetically related lineages, rendering evidence that those mutations are, by all means, only deleterious in the species-specific context. Our results are concordantly indicating that amino acid changes at specific sites are having a strong effect on protein function. Such protein alterations (i.e., change in stability and disease liability) at amino-acid positions that were conserved over large evolutionary timescales might be slightly deleterious or/and counteracted by compensatory changes in the nuclear-coded mitochondrial proteins [47] or may truly reflect adaptation [48, 49].

Hypothesizing that positively selected sites are relevant for adaptation to different climate conditions, we applied statistical models to test this hypothesis. Our PCA of the climate variables were summarized successfully in four (statistically independent) climate factors, and three of them (factor 1, 2, and 4) could be successfully interpreted in climatological terms. Our results showed that climate factors 1, 2 and 3 had a significant effect on the occurrence of certain protein variants of ND4 whereas climate factors 1 and 3 had a significant effect on the occurrence of certain protein variants of Cytb (i.e., amino acid changes at the positively selected sites in ND4 and Cytb). This suggested adaption to climatic/environmental conditions of the OXPHOS genes in the currently studied hares from China. Concordantly, our TreeSAAP analysis for both *ND4* and *Cytb* showed that amino acid changes altered mainly the equilibrium constant (ionization of COOH) property (Table 1). This property was suggested to influence the protein efficiency reducing ROS (reactive oxygen species) production while increasing individual longevity [50]. Romero et al. [51] suggested that alterations in the equilibrium constant allow organisms to better cope with abiotic stress conditions (which could be imposed by ambient climatic conditions). Indeed, the activation of the antioxidant metabolism reducing a ROS excess has been linked to desiccation tolerance in the algae *Mastocarpus* *stellatus* and *Porphyra* *columbina* occurring in the upper intertidal zone [52]. Since abiotic stress in general is linked to metabolic activity and ROS production [51, 53], this directly affects the distribution of a biological species and its success to occupy new ecological niches. Moreover, increased metabolic efficiency has been also related to the capability of diverse animals to invade new ranges [51] and George and Blieck [54] detected significant changes in the equilibrium constant (ionization of COOH) property affecting similar regions in the genes of amphibians, lungfishes, and coelacanths which was suggested as an adaptation to increased oxygen levels and changing metabolic requirements.

Positive selection on diverse mtOXPHOS genes has been suggested by in silico analyses in a wide range of animal species in the contexts of varying ecological and climate conditions. However, only few experimental studies were able to assess the adaptive value of mtDNA variations. A clinal variation of mitochondrial mitotypes along temperature gradients and associations between mitotype and climate have been observed for numerous metazoan species, including humans [18, 55]. Experiments in invertebrates have demonstrated directly that different mitotypes can alter temperature tolerance [56, 57] and that the mitotype was associated with adaptation to temperature in natural environments [18, 58]. Recently, Lajbner et al. [17] used laboratory-based experimental evolution in the fruit fly, *Drosophila* *melanogaster*, to test whether thermal selection could shift population frequencies of two mtDNA haplogroups whose natural frequencies exhibit clinical associations with latitude along the Australian east-coast. They found experimental evidence that the thermal regime in which the laboratory populations were maintained drove changes in haplogroup frequencies across generations. The authors suggested that adaptation to novel environments might routinely involve selection of mitochondrial polymorphisms that optimize thermal performance in those environments, and this process might be relevant to all metazoans, both poikilothermic and homeothermic, and indeed to all eukaryote life [17].

## Conclusion

This study on various hare (genus *Lepus*) species from China provides new evidence for positive selection in ten codons within two mtDNA protein-coding genes (*ND4*, *Cytb*) belonging to OXPHOS complexes I and III while most codons were under purifying selection. Our analyses of the amino acid substitution candidates for positive selection as identified by diverse molecular-statistical test approaches suggested an important impact in protein functions confirming the adaptive implications of these changes. This adaptive variation of amino acid changes was probably driven by environmental conditions as suggested by linear models including climate parameters. The presence of likely introgressed mtDNA in several individuals of some species did, however, not affect statistically the occurrence of protein variants of ND4 and Cytb, somewhat contrary to the hypothesis of Melo-Ferreira et al. [26], who hypothesized possible selective advantages of mtDNA introgressed in some hare species. Furthermore, based on our statistical modeling results the currently observed signals of positive selection are independent from the diverse evolutionary lineages represented by the different species studied.

## Methods

### Sequence collection

Data from three mitochondrial genes *COX1*, *Cytb*, and *ND4*, from 116 individuals covering eight Chinese hare species (*L.* *hainanus*, *L.* *oiostolus*, *L.* *comus*, *L.* *mandshuricus*, *L.* *timidus*, *L.* *capensis*, *L.* *yarkandensis*, *L.* *sinensis*) have been retrieved from Genbank [19]. However, we would like to stress that the evolutionary position and systematics of Chinese hares traditionally considered “*Lepus* *capensis*” and their taxonomy is still under debate [e.g., 59]. In the absence of comprehensive and conclusive population genetic and molecular systematic data, particularly including the forms/taxa “*L.* *tolai*” and “*L.* *tibetanus*”, we leave the name “*L.* *capensis”* as dedicated to the specimens’ sequences submitted to GenBank by the original authors, to avoid potential confusion of sequence identities. Nevertheless, we would like to emphasize that those hares from China termed currently “*L.* *capensis”* appear evolutionarily quite different from nominal cape hares, *Lepus* *capensis* *capens*is, from the Fynbos biome in South Africa [60] as suggested Lado et al. [61].

### Selection analysis

Evidence of mtDNA recombination in animals was demonstrated in several species including mammals [62, 63]. Such recombination events might influence selection detection. Indeed, simulation studies (see Arenas and Posada [64] for an overview) suggested that the likelihood ratio tests (LRTs) were robust to low levels of recombination, but favored the spurious inference of selection when recombination was large and that the number of false positively selected sites has increased as a function of the amount of recombination simulated. Therefore, prior to selection analyses the GARD method implemented on the DATAMONKEY web server (http://www.datamonkey.org/) [65] was employed to search for possible recombination partitions. However, no recombination events were detected in our sequences.

Selection at specific amino acid positions was assessed by comparing the number of non-synonymous substitutions per non-synonymous sites (dN) to numbers of synonymous substitutions per synonymous sites (dS) using the PAML 4 package [66] with the maximum likelihood method. To perform the analyses, a maximum likelihood tree obtained from MEGA version 6 [67] using the best fitting model was used for each gene analyzed (HKY + G for *COX1* (G = 0.16) and *Cytb* (G = 0.24); TN93 + I for *ND4* (I = 0.65)). Six different models proposed by Yang et al. [68] were compared: M0 (one ω ratio), M1a (nearly neutral), M2a (positive selection), M3 (discrete), M7 (beta) and M8 (beta & ω). Pairwise comparisons were performed using Likelihood-ratio tests (LRT) (see [68] for more details).

We further used four additional codon models implemented on the DATAMONKEY web server (http://www.datamonkey.org/) [65] to assess codons under positive or purifying selection: Single Likelihood Ancestral Counting (SLAC), Fixed Effects Likelihood (FEL), Fast Unconstrained Bayesian AppRoximation (FUBAR) and Mixed Effects Model of Evolution (MEME) [69, 70]. In the above cited tests the GTR model was used as the best codon-based substitution model for the three coding genes as directly estimated on the DATAMONKEY web server. Neighbor-Joining trees used for the calculation were automatically generated with DATAMONKEY using the GTR substitution model.

Finally, TreeSAAP [71], which takes into account the magnitude of the impact of the amino acid replacements on local physicochemical properties, was also applied to the current data set. Radical magnitudes of changes ≥ 6, with P value ≤ 0.001, were considered as indicating directional positive selection for a given physicochemical property.

### Protein structure analyses

In order to complement our analyses with the spatial position of sites under positive selection in a tri-dimensional space, we modeled the 3D structures of proteins for which evidence of positive selection was detected, using the SWISS-MODEL server (https://swissmodel.expasy.org/, latest accessed on 22 August 2019, [72]) with default parameters. The stereochemical quality of the models was evaluated using the PROCHECK [73], while the compatibility of an atomic model (3D) with its own amino acid sequence (1D), to assess the 3D protein structure, was evaluated by the ERRAT [74] and VERIFY 3D programs [75] from the UCLA-DOE server (http://www.doembiucla.edu/Services, latest accessed on 22 August 2019). The superimposition, visualization and manipulation of the 3D structures were performed with PYMOL software version 1.5.0.4 [76].

In order to localize positively selected sites in functionally important regions, secondary structures of the obtained protein models were predicted using the PSIPRED server (http://bioinf.cs.uci.ac.uk/psipred/; [77]) and the positions of transmembrane (TM) helices were predicted using the MEMSAT-SVM [55] and MEMEMBED [78] servers (http://bioinf.cs.uci.ac.uk/psipred/). The MEMSAT-SVM server was also used to predict pore-lining regions in transmembrane protein sequences. Moreover, the protein contact Atlas server (https://www.mrc-lmb.cam.ac.uk/rajini/index.html) was used to detect positively selected sites that might be involved in interactions between subunits.

In the obtained models, mutation effects were evaluated by analysis and prediction of protein stability changes upon mutation using the DYNAMUT web server (http://biosig.unimelb.edu.au/dynamut/, [79]). This program can be used to analyze and visualize protein dynamics by sampling conformations and assess the impact of mutations on protein dynamics and stability resulting from vibrational entropy changes.

Furthermore, to assess potential functional effects of the nonsynonymous substitutions, the Protein Variation Effect Analyser (PROVEAN: http://provean.jcvi.org/index.php, [80]; latest accessed on 23 August 2019) was used. PROVEAN evaluates protein sequence variation in an evolutionary context and predicts if an amino acid replacement is likely to have an effect on the protein function. The default confidence threshold of − 2.5 was used to determine, if an amino acid replacement is likely to have an effect on the protein function. The reconstructed ancestral sequence for each locus, using FASTML (http://fastml.tau.ac.il/), of all *Lepus* sequences currently studied was used as a template for DYNAMUT and PROVEAN, and every fixed amino acid replacement per lineage was used as a query.

### Testing for effects of climate variables and trans-specific introgression on positively selected sites

Ambient temperature, among other factors, has been suggested as a possible force driving positive selection on mtOXPHOS genes [6, 8, 9, 31;32]. Therefore, we used the statistical software package R 2.15.0 [R Development Core Team, 81] to run multinomial models for the occurrence of *ND4* protein variants and logistic models for the occurrence of *Cytb* protein variants, to test for effects of climate data on candidate sites for positive selection (and on their respective resultant protein variants). The tested protein variants were based only on positively selected sites as indicated by more than one test in DATAMONKEY and in PAML (see Table 1 in “Results” section). Due to the absence of significant positive selection test results for *COX1* sequences, no linear modeling was performed for that locus. The bioclimatic data were obtained from the WORLDCLIM data set for 2.5 min intervals (Version 1.4, http://www.worldclim.org/bioclim.htm). Nineteen bioclimatic variables were automatically extracted using DIVA-GIS ver. 7.5. Given that particularly high correlations between climatic variables would impose a multicollinearity problem for the linear modelling and to avoid over-parameterization of the models, we first applied an unrotated correlation-matrix based principal components analysis (PCA) on the ln-transformed climate variables, using the SPSS 24.0 statistical software. Ln-transformations of the initial variable values were carried out to reduce their variances, which is recommended for PCA. The resultant climate factors (principal components 1 to 4) explained most (94.7%) of the initially observed variable variance. The summary results and interpretation of the resultant principal components (factors) appear in the “Results” section.

The model syntaxes were based on the following global models (using the package mlogit):

1. m = multinom (*ND4* positively selected site label ~ climate fac.1 + climate fac. 2 + climate fac. 3 + climate fac. 4 + cytb positively selected site label + introgression, random =  ~ species, data = dat),
2. m = glmer((cytb posititively selected site label -1) ~ climate fac.1 + climate fac. 2 + climate fac. 3 + climate fac. 4 + introgression + (1|spec), data = dat, family = binomial) binomial GLMM

where “*ND4* positively selected site label”, as dependent variable, is one among 42 observed protein variants (however, only nine variants were considered and the other variants of relative rare occurrence were excluded, in order not to inflate the degrees of freedom in the models); “cytb positively selected site label” is the respective *Cytb* protein variable co-occurring in the considered individuals, respectively; “climate fac. 1–4” are the respective individual principal component scores resulting from the PCA: “introgression” classifies, whether or not the respective individual sequence has been identified as introgressed in an other species (as stated in the respective publications associated with the respective submitted sequences); and “species”, as random variable, is one of the considered species, to account for potential species-specific effects of occurrence of protein variants (e.g., by unknown mitogenomic interaction). In the *Cytb* models, however, we could not account for the respective ND4 protein variants co-occurring in the considered individuals, due to their big number, which would have lead to an overparameterization of the models.

The modeling results and conclusions were based on the information-theory based approach of model averaging, specifically the relative variable importance values (RVI) that reflect the probabilities of a certain variable occurring in the most likely model. RVI values above 0.7 are considered as “statistically meaningful” [82].

## Data Availability

The genetic datasets used in the current study are freely available via GenBank.

## Abbreviations

ATP6: ATP synthase subunit 6
BEB: Bayes Empirical Bayes
COX1: Cytochrome oxidase 1
Cytb: Cytochrome B
dN: The number of non-synonymous substitutions per non-synonymous sites
dS: Numbers of synonymous substitutions per synonymous sites
FEL: Fixed Effects Likelihood
FUBAR: Fast Unconstrained Bayesian AppRoximation
GARD: Genetic Algorithm for Recombination Detection
GTR: General Time Reversible
LHON: Leber Hereditary Optic Neuropathy
LRT: Likelihood ratio test
MEME: Mixed Effects Model of Evolution
mtDNA: Mitochondrial DNA
ND2: NADH dehydrogenase subunit 2
ND4: NADH dehydrogenase subunit 4
OXPHOS: Oxidative phosphorylation
PCA: Principal components analysis
ROS: Reactive oxygen species
SLAC: Single Likelihood Ancestral Counting
